# Large Interruptions of GAA Repeat Expansion Mutations in Friedreich Ataxia Are Very Rare

**DOI:** 10.3389/fncel.2018.00443

**Published:** 2018-11-21

**Authors:** Sahar Al-Mahdawi, Heather Ging, Aurelien Bayot, Francesca Cavalcanti, Valentina La Cognata, Sebastiano Cavallaro, Paola Giunti, Mark A. Pook

**Affiliations:** ^1^Ataxia Research Group, Division of Biosciences, Department of Life Sciences, College of Health and Life Sciences, Brunel University London, Uxbridge, United Kingdom; ^2^Synthetic Biology Theme, Institute of Environment, Health and Societies, Brunel University London, Uxbridge, United Kingdom; ^3^Ataxia Centre, Department of Molecular Neuroscience, Institute of Neurology, University College London, London, United Kingdom; ^4^Institute of Neurological Sciences, National Research Council, Mangone, Italy; ^5^Institute of Neurological Sciences, National Research Council, Catania, Italy

**Keywords:** Friedreich ataxia, FRDA, frataxin, GAA repeat mutation, GAA repeat interruptions, trinucleotide repeat expansion disease

## Abstract

Friedreich ataxia is a multi-system autosomal recessive inherited disorder primarily caused by homozygous GAA repeat expansion mutations within intron 1 of the frataxin gene. The resulting deficiency of frataxin protein leads to progressive mitochondrial dysfunction, oxidative stress, and cell death, with the main affected sites being the large sensory neurons of the dorsal root ganglia and the dentate nucleus of the cerebellum. The GAA repeat expansions may be pure (GAA)_n_ in sequence or may be interrupted with regions of non-GAA sequence. To our knowledge, there has been no large-scale study of FRDA patient DNA samples to determine the frequency of large interruptions in GAA repeat expansions. Therefore, we have investigated a panel of 245 Friedreich ataxia patient and carrier DNA samples using GAA repeat PCR amplification and *Mbo*II restriction enzyme digestion. We demonstrate that the vast majority (97.8%) of Friedreich ataxia GAA repeat expansion samples do not contain significant sequence changes that would result in abnormal *Mbo*II digestion profiles, indicating that they are primarily pure GAA repeats. These results show for the first time that large interruptions in the GAA repeats are very rare.

## Introduction

Friedreich ataxia (FRDA) is a multi-system autosomal recessive inherited disorder characterized by neurological features of ataxia, dysarthria, weakness, ocular fixation instability, deep sensory loss, and visual and hearing impairment, together with non-neurological features such as hypertrophic cardiomyopathy, diabetes mellitus, kyphoscoliosis, and foot deformities (Reetz et al., 2015). The mean age of onset of FRDA is 15 years, with most cases developing by age 25, although rare cases of late onset FRDA (26–39 years), or very late onset FRDA (40 years or over) have been reported (Durr et al., 1996; Filla et al., 1996). FRDA is primarily caused by homozygous GAA trinucleotide repeat expansion mutations within intron 1 of the frataxin (*FXN*) gene, leading to deficit of the essential mitochondrial protein frataxin. The resulting deficiency of frataxin protein leads to progressive mitochondrial dysfunction, oxidative stress, and cell death, with the main affected sites being the large sensory neurons of the dorsal root ganglia and the dentate nucleus of the cerebellum (Campuzano et al., 1996, 1997). The number of GAA repeats in unaffected individuals generally ranges from 6 to 27 repeats, although rare cases of 33 to 130 repeats have been identified (Cossee et al., 1997; Montermini et al., 1997; Ohshima et al., 1999). In contrast, approximately 96% of FRDA patients have homozygous GAA repeat expansions ranging from 44 to 1,700 repeats, with 600–900 GAA repeats being the most common. The remaining FRDA patients are compound heterozygous for a GAA repeat expansion and a second *FXN* mutation. The age of onset is inversely correlated with the GAA repeat number, particularly for the shorter allele (GAA1), with a prediction of 2.3 years earlier onset for every 100 GAA repeats added to GAA1 (Reetz et al., 2015). Thus, the GAA1 allele is considered to give the closest genotype–phenotype relationship. However, the GAA repeat size accounts for only approximately 36% to 56% of the variation in age of onset (Durr et al., 1996; Filla et al., 1996; Reetz et al., 2015). This suggests that there are other contributory mechanisms such as somatic mosaicism, interruptions in the GAA repeat sequence, and other modifying genes or environmental factors, which influence age of onset (Filla et al., 1996; Pandolfo, 2009; Reetz et al., 2015). Earlier age of onset increases the frequency and severity of neurological and non-neurological symptoms. In contrast, patients with late-onset disease have a milder phenotype and slower disease progression (Durr et al., 1996).

In the majority of cases of FRDA, the GAA repeat expansion mutations have been characterized only in terms of the overall repeat size rather than sequence content, which can be either pure GAA or interrupted GAA. Where full sequencing of *FXN* GAA repeats has been possible due to comparatively short repeats (up to approximately 130 repeats), there are reports of interrupted GAA repeat expansion sequences, such as (GAGGAA)_5-9_ or (GAAGGA)_65_, and these are associated with either absence of FRDA disease phenotype (Cossee et al., 1997; Montermini et al., 1997; Ohshima et al., 1999) or atypical mild late-onset or very late-onset FRDA disease phenotype (Cossee et al., 1997; Epplen et al., 1997; Moseley et al., 1998; McDaniel et al., 2001; Sharma et al., 2004; Stolle et al., 2008). *In vitro* studies have shown that interrupted GAA repeats inhibit triplex formation, alleviate transcription inhibition, and reduce repeat instability (Ohshima et al., 1999; Sakamoto et al., 2001). Therefore, it is thought that interruptions in the GAA repeat sequence can result in maintenance of *FXN* expression levels and reduced somatic instability of GAA repeats when compared to similar sized pure GAA repeats, thereby impacting upon FRDA disease progression. Further studies have shown that pure GAA repeats are stable up to a threshold of 44 repeats, after which they become unstable (Sharma et al., 2004). In a study of FRDA carriers, 107 pure GAA repeats were unstable, whereas 114 interrupted GAA repeats were stable by small-pool PCR of blood samples (Pollard et al., 2004). Also a (GAA)_90_(GAAAGAA)_9_(GAA)_20_ interrupted 112 GAA repeat has been shown to stably transmit through two generations (Cossee et al., 1997). Finally, somatic instability of the GAA1 allele has been identified in two FRDA patients who have one large GAA2 allele and a small GAA1 allele of either 44 or 66 repeats, while their sibling with one large GAA2 allele and one somatically stable GAA1 allele of 37 repeats was clinically normal (Sharma et al., 2004). So, it would appear that somatic instability may have a significant role to play in FRDA disease progression and interrupted GAA repeats are likely to confer some protection in the form of somatic stability and maintenance of *FXN* expression levels.

Analysis of the much more frequent large GAA2 alleles (>130 repeats) for the presence or absence of interruptions has not previously been performed to any great extent, primarily because of the technical difficulty in obtaining accurate sequence of an entire long GAA repeat expansion. Sakamoto et al. (2001) sequenced 11 expanded FRDA alleles for as long as was technically possible, up to approximately 200 repeats, and they identified interruptions in 5 of the 11 samples, which were clustered within the last 10–15 repeats at the 3′ end. They also used *Ear*I (GAAGAG recognition sequence) and *Mnl*I (GAGG recognition sequence) restriction enzyme digestion to identify specified interruptions within 4 out of 22 FRDA patient samples (Sakamoto et al., 2001). Subsequently, Holloway et al. (2011) reported the development of a useful method to determine non-specified GAA repeat interruptions comprising long-range GAA PCR followed by *Mbo*II (GAAGA recognition sequence) restriction enzyme digestion. Only four FRDA patients were investigated in this study, but one of the four was found to contain an extensive region of GAA repeat interruptions in their larger 1140 GAA repeat allele, which was partially sequenced to be (GAA)_21_(GGAGAA)_5_(GGAGGAGAA)_70_(GAA)_n_. To expand upon these previous small-number studies, we have now addressed the question, “Do most FRDA patients have pure GAA repeat expansions?” by investigating a large panel of 245 FRDA patient and carrier DNA samples using long-range GAA repeat PCR amplification and *Mbo*II restriction enzyme digestion. We find that the vast majority (97.8%) of FRDA patient DNA samples do not contain substantial sequence changes that would result in abnormal *Mbo*II digestion profiles, indicating that they are primarily pure GAA repeats. We discuss the implications of this finding with regard to GAA repeat instability and FRDA disease progression.

## Materials and Methods

### *Mbo*II Digestion Analysis

We obtained 245 peripheral blood genomic DNA samples from FRDA patients (238 samples) and carriers (7 samples) that had previously undergone GAA repeat expansion size determination (Supplementary Table S1) (ethics approval granted within the European Union Seventh Framework Programme [FP7/2007-2013] under grant agreement number 242193/EFACTS). We also obtained genomic DNA samples from cerebellum autopsy tissues from three FRDA patients (tissues were registered with the HTA under Brunel University Licensing number 12543) and five ear biopsies from previously described GAA repeat expansion-based Y47R and YG8sR FRDA mouse models (Anjomani Virmouni et al., 2015) (animal procedures were carried out in accordance with the UK Home Office “Animals (Scientific Procedures) Act 1986” and with approval from the Brunel University London Animal Welfare and Ethical Review Board). We then performed long-range PCR of the samples (approximately 100 ng input DNA) using either the Expand High Fidelity PCR System, dNTPack (Roche), or the Long Range PCR Kit (Qiagen) together with GAA-B-F (5′-AATGGATTTCCTGGCAGGACGC-3′) and GAA-B-R (5′-GCATTGGGCGATCTTGGCTTAA-3′) primers as previously described (Holloway et al., 2011). The thermocycling conditions used were (i) Roche Kit: 94°C for 2 min; 10 cycles of 94°C for 10 s, 60°C for 30 s, 68°C for 45 s; 20 cycles of 94°C for 10 s, 60°C for 30 s, 68°C for 1 min with 20 s increments; and a final cycle of 68°C for 10 mins, or (ii) Qiagen Kit: 93°C for 3 min; 35 cycles of 93°C for 15 s, 62°C for 30 s, 68°C for 5 min, and a final cycle of 68°C for 10 min. The amplified PCR products contained the GAA trinucleotide repeat expansion with flanking sequences of 170bp at the 5′ end and 120bp at the 3′ end. A 5 μl sample of each PCR product was checked by running on 1% agarose gels. The positive samples were then digested with *Mbo*II, which has a cleavage sequence of 5′-GAAGA(8/7)-3′. PCR sample (17 μl) was digested in a total reaction volume of 20 μl at 37°C for 1 h. Digested DNA fragments were subjected to heating at 95°C for 10 min followed by slow cooling to room temperature to prevent potential heteroduplex formation, then separated by running on 2% agarose gels [1% Nusieve (Seakem Agarose GTG) and 1% Metaphor agarose]. As the *Mbo*II restriction enzyme cuts at the recognition site 5′-GAAGA (8/7)-3′, pure GAA repeats are completely cut by *Mbo*II leaving only two fragments from the uncut flanking sequences, 171/170bp upstream (designated hereafter as “170bp”) and 117/118bp downstream (designated hereafter as “120bp”), which do not contain *Mbo*II sites. On the other hand, if the GAA repeat expansion contains interrupted GAA sequences that are not cut by *Mbo*II, a different pattern of bands would be obtained upon agarose gel electrophoresis: either two bands with sizes that differ from the expected 170 and 120bp or else more than two bands.

### DNA Sequencing

GAA PCR products were purified from agarose gels using Geneclean (MP Biomedicals), then cloned into pCR4.0 using the TOPO TA cloning kit (Invitrogen). For each PCR product, plasmid DNA from two independent colonies was Sanger sequenced by Genewiz using T3 and T7 sequencing primers.

### Statistical Analysis

Correlation and regression analysis of GAA repeat sizes versus age of onset and subsequent ANOVA statistics were performed using Microsoft Excel data analysis tools (GAA sizes for samples 89–245 were obtained from the EFACTS database).

## Results

### The Majority of FRDA GAA Repeats Do Not Contain Interruptions Detected by *Mbo*II Analysis

**FIGURE 1:**
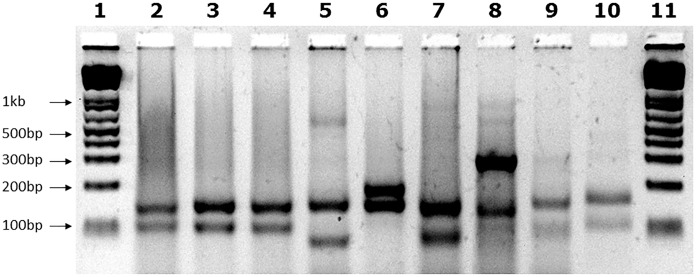
*Mbo*II digest results. Agarose gel showing *Mbo*II digests of GAA PCR products of FRDA samples. The expected 170bp (5′) and 120bp (3′) undigested GAA-flanking fragments from normal pure GAA repeat expansion FRDA samples are shown in lanes 2, 3, and 4. These band sizes can be seen in between the 200 and 100bp fragments of the 1 Kb+ DNA ladder markers, which are loaded into lanes 1 and 11 of the gel. Lane 5 shows a large *Mbo*II band of approximately 600bp that was obtained from the positive interrupted GAA repeat sequence from the “NEP” BAC transgenic mouse that contains approximately 500 triplet repeats with the previously determined interrupted sequence of (GAA)_21_(GGAGAA)_5_(GGAGGAGAA)_70_(GAA)_n_ (Holloway et al., 2011). In addition for this positive sample, we also identified the expected 5′ flanking band of 170bp, together with a smaller band of less than 100bp that we sequenced and we showed to contain a 27bp deletion in the 3′ flanking region. Lane 6 shows an abnormal band of 200bp representing the 80bp duplication in the 3′ GAA flanking region. Lane 7 shows an abnormal band of approximately 100bp representing the 19bp deletion in the 3′ GAA flanking region. Lanes 8, 9, and 10 contain abnormal bands of approximately 300, 100, and 180bp, respectively, that are likely to contain a region of interrupted GAA repeat sequence within the body of one or other of the large FRDA GAA repeat expansions.

We examined the GAA repeat status in 245 FRDA patient and carrier peripheral blood DNA samples by long-range GAA PCR followed by *Mbo*II restriction enzyme digestion and agarose gel electrophoresis. This method can detect significant GAA interruptions in either of the two alleles at a resolution of approximately 20bp added to either of the two expected *Mbo*II bands of 170 and 120bp or approximately 50bp of internal interruptions (Holloway et al., 2011). As a positive control for known interrupted *FXN* GAA repeat sequence, we used DNA from the “NEP” BAC transgenic mouse that contains approximately 500 triplet repeats with the previously determined interrupted sequence of (GAA)_21_(GGAGAA)_5_(GGAGGAGAA)_70_(GAA)_n_ (Holloway et al., 2011). Eighteen of our FRDA DNA samples failed to generate sufficient PCR product for the assay, leaving 227 samples for further analysis; 220 FRDA patients and 7 carriers (Table 1). 222 out of these 227 samples (97.8%) produced only the expected two *Mbo*II bands of 170 and 120bp, indicating the likely presence of pure GAA repeats in both alleles in the vast majority of cases (Figure 1, lanes 2–4). However, the remaining five samples (2.2%) showed abnormal *Mbo*II band profiles, indicating disruption of at least one of the two alleles. One sample produced *Mbo*II bands of 170 and 200bp, indicating an insertion of approximately 80bp in the 3′ GAA flanking sequence (Figure 1, lane 6). Sequencing of the cloned GAA PCR products from this sample confirmed that there was an 80bp duplication in the 3′ GAA flanking region of the smaller allele, duplicating the sequence 24–104bp downstream of the final GAA repeat, that did not involve any interruption of the GAA repeat itself. Another sample produced *Mbo*II bands of 170 and 100bp, indicating a deletion of approximately 20bp in the 3′ GAA flanking sequence (Figure 1, lane 7). Sequencing of the cloned GAA PCR products from this sample confirmed that there was a 19bp deletion of the smaller allele immediately after the GAA repeat sequence, but not involving any interruption of the GAA repeat itself. The remaining three abnormal *Mbo*II samples produced bands of approximately 100, 180, and 300bp (Figure 1, lanes 8–10), but sequencing did not identify any substantial insertions or deletions in the 5′ or 3′ flanking regions or in the GAA repeats close to these flanking regions, indicating the likely presence of significant GAA repeat interruptions within the body of the GAA repeat expansion alleles and beyond the capability of our sequencing technology. However, sequencing of up to 56 repeats at the 5′ and 3′ ends of the nine FRDA samples shown in Figure 1 did reveal small GAA repeat sequence interruptions in three of the nine samples (i.e., 33%) (Figure 1, lane 7 sample: …(GAA)_23_A(GAA)_5_AGAA, lane eight sample:…(GAA)_26_A(GAA)_4_G(GAA)_2_ and lane 10 sample: …(GAA)_4_GAG(GAA)_5_. These interruptions were all within the last seven repeats at the 3′ end of the GAA repeat sequence, in a similar location and at a similar frequency to the small 3′ GAA repeat interruptions that were detected in 5 out of 11 (i.e., 45%) FRDA samples as previously reported by Sakamoto et al. (2001).

**Table 1:** Summary of *Mbo*II digestion results.

*Mbo*II digestion results	Total numbers	Patient numbers	Carrier numbers
Total no. of samples	245	238	7
Total no. of *Mbo*II digestions	227	220	7
170 and 120bp *Mbo*II bands only	222	215	7
Other sizes of *Mbo*II bands	5	5	0
Confirmed insertion in 3′ flanking region	1	1	0
Confirmed deletion of 3′ flanking region	1	1	0
Unknown change	3	3	0

### Confirmation of the Inverse Correlation Between GAA Repeat Size and Age of Disease Onset

**FIGURE 2:**
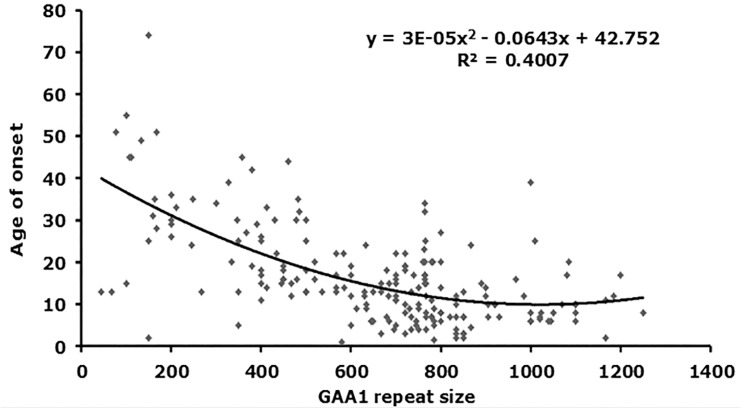
Correlation analysis of GAA repeat size with age of onset. A graph representing GAA1 repeat size v age of onset is shown (*n* = 199). The best fit was for a quadratic model, for which the equations and *R*^2^-values are shown (ANOVA *P*-values are listed in Table 2).

From our starting cohort of 245 samples, we identified 199 homozygous GAA repeat expansion FRDA patient samples for which data were available on age of onset in addition to both GAA repeat allele sizes (Supplementary Table S1). In agreement with previous studies (Durr et al., 1996; Filla et al., 1996; Reetz et al., 2015), our analysis of all 199 samples revealed a strong inverse correlation between the size of the GAA1 allele and age of onset, with a Pearson’s correlation coefficient, *R* = −0.58, and *R*^2^ = 0.34, using a linear model, and an even stronger correlation, *R* = −0.63, and *R*^2^ = 0.40, using a quadratic model (Figure 2 and Table 2). This would suggest that up to 40% of the variation in age of onset in our sample collection is determined by GAA1 repeat expansion length. The correlations for either the larger GAA2 allele repeat size or the average GAA repeat size with age of onset were also significant, but to a lesser degree (*R*^2^ = 0.30 and 0.12, respectively, using a quadratic model, Table 2). The five samples that showed abnormal *Mbo*II band profiles came from FRDA patients with ages of onset ranging from 5 to 45 years and, therefore, did not reveal any unusual age of onset subgrouping.

**Table 2:** Summary of GAA repeat size versus age of onset correlation and regression analysis.

Samples	*R* (linear)	*R*^2^ (linear)	*R* (quadratic)	*R*^2^ (quadratic)	*P*-value	*n*
All GAA1	−0.58	0.34	−0.63	0.40	<0.0001	199
All GAA2	−0.28	0.08	−0.35	0.12	<0.001	199
All average GAA	−0.51	0.26	−0.55	0.30	<0.0001	199

### Analysis of GAA Repeat Expansion Dynamics

GAA repeat expansions are known to be dynamic entities, exhibiting both intergenerational and somatic instability of repeat lengths. However, it is possible that GAA repeat expansions may also vary at either intergenerational or somatic levels according to sequence content (pure GAA or interrupted repeat sequence). To address potential intergenerational repeat changes, we identified four examples of carrier parents and FRDA-affected offspring within our collection of FRDA patient and carrier DNA samples (Supplementary Table S1) and examined these samples by *Mbo*II digestion analysis. We found that for all four examples only the expected two *Mbo*II bands of 170 and 120bp were produced in both parents and offspring, indicating primarily pure GAA repeats. This indicates that no gross GAA repeat interruptions detectable by *Mbo*II digestion occurred across generations.

**FIGURE 3:**
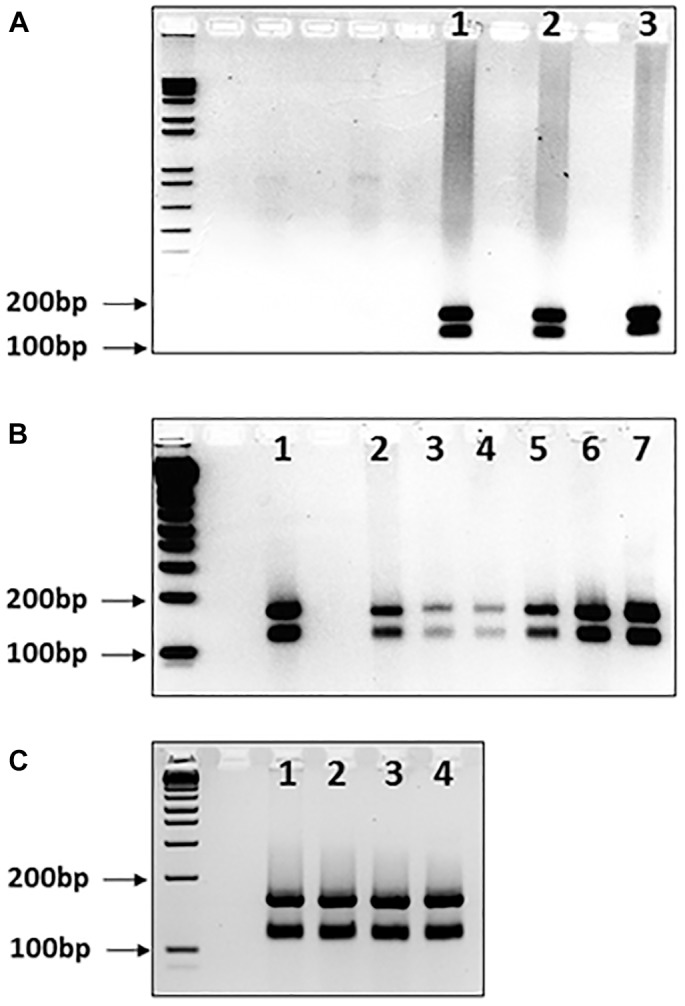
*Mbo*II digests of GAA repeat expansions from human FRDA somatic tissues and mouse FRDA intergenerational and somatic tissues. Agarose gels showing *Mbo*II digests of GAA PCR products of **(A)** FRDA patient cerebellum tissue samples, **(B)** YG8sR mouse ear biopsy samples and human FRDA blood samples, and **(C)** four tissues from one YG8sR mouse. In each case, the expected 170 and 120bp undigested GAA-flanking fragments can be identified in between the 200 and 100bp fragments of the 1 Kb+ DNA ladder marker, which is loaded into the first lane of each gel. **(A)** Lanes 1–3 show the results from cerebellum tissue samples from three FRDA patients. **(B)** Lanes 1 and 2 are from FRDA patient blood samples; lanes 3–6 are from ear biopsy samples from 4 GAA repeat expansion-based YG8sR mice of four different generations, and lane 7 is from an ear biopsy sample from the Y47R mouse which has nine GAA repeats. **(C)** Lanes 1–4 are from brain, cerebellum, heart, and liver tissues of the YG8sR mouse, respectively.

Another possible occurrence is that pure GAA sequences may develop interruptions in different somatic tissues. We addressed this issue by determining the *Mbo*II digestion profiles from cerebellum autopsy tissue samples from three FRDA patients, since cerebellum tissue is known exhibit a high degree of GAA repeat instability in FRDA (Al-Mahdawi et al., 2004). Pure GAA repeat expansions were identified for each sample, indicating that, at least in these cases, there was no evidence of tissue-specific differences of large GAA repeat interruptions that could be detected by *MboII* analysis (Figure 3A, lanes 1–3).

Our data obtained from human samples is supported by similar *Mbo*II digestion studies of GAA repeat expansion-based FRDA YAC transgenic mice. We analyzed DNA samples from four generations of YG8sR FRDA mice, which have previously been shown to contain 120 pure GAA repeats (Anjomani Virmouni et al., 2015). The expected two *Mbo*II bands of 170 and 120bp were obtained for all samples, indicating intergenerational transmission of pure GAA repeats as detected by *Mbo*II analysis (Figure 3B, lanes 3–6). Furthermore, the expected two *Mbo*II bands of 170 and 120bp were obtained for samples of brain, cerebellum, heart, and liver tissues from the same 1-year-old YG8sR FRDA mouse, indicating somatic stability of pure GAA repeats as detected by *Mbo*II analysis (Figure 3C, lanes 1–4).

## Discussion

To our knowledge, there has been no large-scale study of FRDA patient DNA samples to determine the frequency of large interruptions in GAA repeat expansions. Therefore, we aimed to address the question, “Do most FRDA patients have mainly pure GAA repeat expansions?” by performing long-range GAA repeat PCR amplification and *Mbo*II restriction enzyme digestion upon a large panel of 245 FRDA patient and carrier DNA samples. We found that the majority of GAA repeats from FRDA patient and carrier samples do not show significant extensive sequence changes that would result in abnormal *Mbo*II profiles, indicative of primarily pure GAA repeats throughout most of the repeat length. So the answer to our question is, yes, most FRDA patients have mainly pure GAA repeat expansions. However, we also confirmed the previous finding by Sakamoto et al. (2001) that a significant number of FRDA samples contain small sequence interruptions localized to the 3′ end of the GAA repeats. These small localized repeat interruptions would not be detected by the *Mbo*II enzymatic digestion method and they would not be expected to have any major impact upon GAA repeat expansion dynamics in the way that a large *Mbo*II-detected interruption may have.

We recognize that there are some limitations to the *Mbo*II digestion method of analysis. Firstly, *Mbo*II digestion does not identify sequence at the base pair level. Sequencing of long homozygous GAA repeats, which can be up to 1,700 repeats in size, is technically still too difficult to perform. However, sequencing of small GAA repeat alleles is possible after separation from larger GAA repeat alleles by gel purification techniques. In future, technologies such as GAA TP-PCR and single molecule sequencing may prove useful for more detailed analysis of GAA repeat interruptions in FRDA. TP-PCR studies may allow the detection of non-GAA interruptions within approximately the first 100 repeats at the 5′ or 3′ ends of GAA repeat expansions (Ciotti et al., 2004), although again TP-PCR will not identify sequence at the base pair level. Alternatively, single molecule sequencing has been used successfully for the identification of AGG interruptions in CGG repeats of fragile X syndrome premutation individuals (Ardui et al., 2017). Secondly, the *Mbo*II method will only detect approximately 20bp added to the 170 or 120bp flanking regions or >50bp of internal interruptions in the GAA repeat expansion, but will not detect smaller interruptions or (GAAGA)_n_ interruptions because these will be cut by *Mbo*II. Furthermore, care must be taken to exclude possible heteroduplex formation, which may cause difficulties in interpretation of *Mbo*II digests by producing an apparent third *Mbo*II band that is not real.

Overall, our findings indicate that, since most FRDA patients appear to carry primarily pure GAA repeat expansions throughout most of the length of the repeat, they are likely to exhibit a classical FRDA phenotype, showing well established decreases in frataxin gene expression due to epigenetic changes, R loop and heterochromatin formation, with consequential mitochondrial dysfunction, oxidative stress, and cell death, especially of large sensory neurons (Campuzano et al., 1996; Saveliev et al., 2003; Groh et al., 2014; Abeti et al., 2015, 2016, 2018a,b). Pure GAA repeat expansions are also likely to be unstable, increasing in length in aging somatic tissues, particularly in expansion-susceptible tissues such as the dorsal root ganglia and cerebellum (De Biase et al., 2007), and thereby increasing the severity of FRDA disease progression. In contrast, the lesser number of FRDA patients who may be shown to have *Mbo*II-detected GAA repeat interruptions may have a less progressive frataxin deficit within their cells and thus a less severe FRDA disease progression, which may suggest a prognosis of milder disease. Due to the unstable nature of pure GAA repeat expansions, individuals with pure GAA repeat expansions are likely to transmit even larger GAA repeats to any offspring. However, this may not be the case for individuals with significant interruptions in their GAA repeat expansions, since a (GAAAGAA)_n_ interrupted 112 GAA repeat has been reported to be stably transmitted through two generations (Cossee et al., 1997). Understanding whether potential FRDA carriers in the population have either pure GAA repeats or large interrupted GAA repeats may also be of significance, since large interrupted GAA repeats may block the appearance of pathologically sized GAA repeat expansions.

Our findings concerning the paucity of large GAA repeat interruptions in FRDA also add to the understanding of trinucleotide repeat disorders in general. It is known that interruptions within trinucleotide repeat sequences, such as the (CAG)_n_ *ATXN1* repeat, (CTG)_n_ *DM1* repeat, and (CGG)_n_ *FMR1* repeat, can make them less prone to expansion. Studies in yeast have shown that such interruptions in (CTG)_n_ repeats reduce hairpin formation during DNA replication and thus inhibit expansion rates (Rolfsmeier and Lahue, 2000). Other *in vitro* studies of (CAG)_n_ and (CGG)_n_ repeats have demonstrated inhibitory effects of interruptions on the formation of slipped strand DNA structures, thereby reducing their instability (Pearson et al., 1998). Whatever the exact mechanisms, the interplay between trinucleotide repeat interruptions and stability may have important consequences on related genetic disease phenotype. Thus, the importance of interruptions in (CAG)_n_ repeat expansions with regard to disease pathology has previously been reported for spinocerebellar ataxia type 1 (SCA1) (Menon et al., 2013). Interruptions in the (ATTCT)_n_ repeat have also been shown to modify the SCA10 disease phenotype (Matsuura et al., 2006). To this list, we can now add our findings in FRDA, where large interruptions in the (GAA)_n_ repeat are very rare and therefore are unlikely to significantly modify the age of onset FRDA disease phenotype on an overall cohort basis, but may have significant effects for individual FRDA cases.

Further insights into the relevance of interruptions of trinucleotide repeats and associated genetic diseases may be gained by studying suitable mouse models. For example, the comparison of FRDA and Huntington disease (HD) transgenic and knock-in mice also sheds some light on our understanding of the importance of trinucleotide repeat sequence content, somatic instability, and disease phenotype. Both CAG transgenic and CAG knock-in mice can give rise to HD-like phenotypes. However, both of the HD transgenic mice, BACHD and YAC128, contain interrupted repeats (Pouladi et al., 2012), which in both cases leads to complete somatic stability, whereas the three HD knock-in mice (Shelbourne et al., 1999; Wheeler et al., 1999; Ishiguro et al., 2001) have pure CAG repeats and extensive somatic instability. Further studies of HD transgenic mice with pure CAG repeats and knock-in mice with interrupted repeats should help to sort out the underlying mechanisms here. The same is true for investigations of the GAA repeats of FRDA mouse models. YG8R and KIKO mice have pure GAA repeats and extensive somatic and intergenerational GAA repeat instability with a bias toward expansion, whereas the BAC transgenic mice have extensive interruptions in the GAA repeat sequence and show no somatic instability (Al-Mahdawi et al., 2004). However, this does not translate directly into a significant difference in disease phenotype since all three FRDA mouse models exhibit similarly mild FRDA-like phenotypes (Miranda et al., 2002; Al-Mahdawi et al., 2006). Our reported finding now that YG8sR FRDA YAC transgenic mice contain pure GAA repeats that remain uninterrupted throughout intergenerational transmission and across different somatic tissues means that such mice will be liable to further expansion of the GAA repeat size across generations and within tissues throughout age. It will be interesting to see how increasing GAA repeat sizes correlate with FRDA-like disease severity in these mice.

## Conclusion

We have shown that major interruptions of GAA repeat expansions are very rare, but when they do occur, they may be of importance to FRDA disease progression, in a similar manner to interruptions in other trinucleotide repeat sequences and their associated genetic diseases. In contrast, we have confirmed that small GAA repeat sequence interruptions occur more frequently at the 3′ end of the GAA repeat sequence, but such small interruptions are not expected to have such a major impact on GAA repeat expansion dynamics. Ultimately, future studies that enable GAA repeat expansion sequence verification at the base pair resolution are needed to allow further in depth conclusions to be made regarding the importance of GAA repeat interruptions and FRDA disease.

## Author Contributions

SA-M, HG, AB, FC, VLC, SC, and MP performed the experiments. SA-M, HG, AB, PG, and MP conceived and designed the study. SA-M, HG, PG, and MP wrote the manuscript. All authors read and approved the manuscript.

## Conflict of Interest Statement

The authors declare that the research was conducted in the absence of any commercial or financial relationships that could be construed as a potential conflict of interest.
