# Supplementary material for: Large Interruptions of GAA Repeat Expansion Mutations in Friedreich Ataxia Are Very Rare
Source: Front Cell Neurosci. 2018 Nov 21;12:443. doi: 10.3389/fncel.2018.00443 (PMC6258883; doi:10.3389/fncel.2018.00443)
Supplement: Supplementary file 1 [file Table_1.DOCX]

**Table S1: *Mbo*II digestion results and age of onset data**

| **No.** | **GAA1** | **GAA2** | **120bp and 170bp *Mbo*II bands** | ***Mbo*II comments** | **Age of Onset** |
| --- | --- | --- | --- | --- | --- |
|  |  |  |  |  |  |
| **1** | **1023** | **1258** | **✔** |  | **8** |
| **2 (F1)** | **720** | **760** | **✔** |  | **8** |
| **3 (F1)** | **N C** | **E** | **✔** |  | **ND** |
| **4 (F1)** | **N C** | **720** | **✔** |  | **ND** |
| **5 (F2)** | **N C** | **520** | **✔** |  | **ND** |
| **6** | **N** | **500** | **✔** |  | **7** |
| **7 (F2)** | **720** | **720** | **✔** |  | **22** |
| **8** | **500** | **720** | **✔** |  | **25** |
| **9 (F3)** | **N C** | **900** | **✔** |  | **ND** |
| **10 (F3)** | **N C** | **E** | **✔** |  | **ND** |
| **11 (F3)** | **730** | **1040** | **✔** |  | **9** |
| **12** | **750** | **900** | **✔** |  | **10** |
| **13** | **630** | **730** | **✔** |  | **12** |
| **14** | **630** | **1040** | **✔** |  | **13** |
| **15** | **680** | **840** | **✔** |  | **7** |
| **16** | **763** | **1043** | **✔** |  | **23** |
| **17** | **850** | **1000** | **✔** |  | **3** |
| **18 (F4)** | **N C** | **E** | **✔** |  | **ND** |
| **19 (F4)** | **E** | **E** | **✔** |  | **8** |
| **20** | **567** | **752** | **✔** |  | **17** |
| **21** | **E** | **E** | **✔** |  | **17** |
| **22** | **500** | **730** | **✔** |  | **13** |
| **23** | **E** | **E** | **✔** |  | **8** |
| **24** | **E** | **E** | **✔** |  | **14** |
| **25** | **E** | **E** | **✔** |  | **20** |
| **26** | **765** | **765** | **✔** |  | **34** |
| **27** | **460** | **765** | **✔** |  | **44** |
| **28** | **E** | **E** | **✔** |  | **18** |
| **29** | **765** | **765** | **✔** |  | **32** |
| **30** | **765** | **1100** | **✔** |  | **16** |
| **31** | **430** | **1245** | **✔** |  | **30** |
| **32** | **1250** | **1465** | **✔** |  | **8** |
| **33** | **E** | **E** | **✔** |  | **11** |
| **34** | **782** | **782** | **✔** |  | **20** |
| **35** | **1040** | **1040** | **✔** |  | **6** |
| **36** | **163** | **600** | **✔** |  | **35** |
| **37** | **N C** | **1000** |  | **extra band at 180bp** | **ND** |
| **38** | **760** | **890** | **✔** |  | **20** |
| **39** | **890** | **890** | **✔** |  | **15** |
| **40** | **1045** | **1045** | **✔** |  | **6** |
| **41** | **765** | **1065** | **✔** |  | **17** |
| **42** | **112** | **940** |  | **80bp insertion in 3’ flanking region** | **45** |
| **43** | **905** | **965** | **✔** |  | **7** |
| **44** | **700** | **1040** | **✔** |  | **16** |
| **45** | **900** | **1300** | **✔** |  | **12** |
| **46** | **N** | **330** | **✔** |  | **65** |
| **47** | **930** | **930** | **✔** | **(Fig. 1 lane 2)** | **7** |
| **48** | **350** | **750** | **✔** |  | **5** |
| **49** | **500** | **800** | **✔** | **(Fig. 1 lane 3)** | **30** |
| **50** | **1070** | **1460** | **ND** |  | **10** |
| **51** | **400** | **1000** | **✔** |  | **25** |
| **52** | **633** | **760** | **✔** |  | **24** |
| **53** | **740** | **1200** | **✔** |  | **17** |
| **54** | **700** | **1000** | **✔** |  | **11** |
| **55** | **108** | **1040** | **✔** |  | **45** |
| **56** | **765** | **1045** | **✔** |  | **17** |
| **57** | **1000** | **1000** | **✔** |  | **6** |
| **58** | **1000** | **1000** | **ND** |  | **6** |
| **59** | **780** | **780** | **ND** |  | **20** |
| **60** | **249** | **559** | **✔** |  | **35** |
| **61** | **906** | **906** | **✔** |  | **10** |
| **62** | **536** | **809** | **✔** | **(Fig. 1 lane 4)** | **13** |
| **63** | **N** | **1180** | **ND** |  | **ND** |
| **64** | **150** | **573** | **✔** |  | **74** |
| **65** | **77** | **127** | **✔** |  | **51** |
| **66** | **328** | **1194** | **✔** |  | **39** |
| **67** | **478** | **1257** | **✔** |  | **30** |
| **68** | **358** | **358** | **✔** |  | **45** |
| **69** | **160** | **1040** | **ND** |  | **31** |
| **70** | **696** | **800** | **✔** |  | **4** |
| **71** | **800** | **1013** | **✔** |  | **20** |
| **72** | **766** | **1046** | **✔** |  | **25** |
| **73** | **390** | **390** | **✔** |  | **29** |
| **74** | **N** | **E** | **✔** |  | **63** |
| **75** | **N** | **E** | **✔** |  | **ND** |
| **76** | **765** | **1045** | **ND** |  | **15** |
| **77** | **E** | **E** | **ND** |  | **36** |
| **78** | **485** | **485** | **✔** |  | **32** |
| **79** | **800** | **800** | **✔** |  | **27** |
| **80** | **483** | **905** | **✔** |  | **35** |
| **81** | **E** | **E** | **✔** |  | **20** |
| **82** | **765** | **1100** | **✔** |  | **20** |
| **83** | **700** | **1000** | **✔** |  | **22** |
| **84** | **400** | **400** | **✔** |  | **18** |
| **85** | **1010** | **1207** | **✔** |  | **25** |
| **86** | **1085** | **1165** | **✔** |  | **20** |
| **87** | **1100** | **1400** | **✔** |  | **8** |
| **88** | **347** | **1301** | **✔** |  | **30** |
| **89** | **200** | **200** | **✔** |  | **26** |
| **90** | **1020** | **1220** | **✔** |  | **ND** |
| **91** | **350** | **1020** | **✔** |  | **13** |
| **92** | **E** | **E** | **✔** |  | **ND** |
| **93** | **350** | **885** | **✔** |  | **25** |
| **94** | **850** | **1050** | **✔** |  | **13** |
| **95** | **400** | **1000** | **✔** |  | **15** |
| **96** | **683** | **983** | **✔** |  | **12** |
| **97** | **67** | **1100** | **✔** |  | **13** |
| **98** | **E** | **E** | **✔** |  | **ND** |
| **99** | **600** | **767** |  | **extra band at 300bp** | **5** |
| **100** | **520** | **850** | **✔** |  | **20** |
| **101** | **850** | **1180** | **✔** |  | **7** |
| **102** | **467** | **667** | **✔** |  | **ND** |
| **103** | **967** | **1100** | **✔** |  | **16** |
| **104** | **834** | **1167** | **✔** |  | **7** |
| **105** | **1167** | **1167** | **ND** |  | **2** |
| **106** | **N** | **612** | **✔** |  | **15** |
| **107** | **400** | **534** | **✔** |  | **26** |
| **108** | **667** | **1100** | **ND** |  | **15** |
| **109** | **867** | **1134** | **ND** |  | **4.5** |
| **110** | **150** | **850** | **✔** |  | **2** |
| **111** | **150** | **534** | **✔** |  | **25** |
| **112** | **367** | **1100** | **✔** |  | **27** |
| **113** | **1020** | **1220** | **✔** |  | **7** |
| **114** | **567** | **834** | **✔** |  | **14** |
| **115** | **1000** | **1000** | **✔** |  | **39** |
| **116** | **1100** | **1200** | **ND** |  | **10** |
| **117** | **450** | **720** | **✔** |  | **16** |
| **118** | **685** | **920** | **✔** |  | **15** |
| **119** | **1067** | **1167** | **ND** |  | **ND** |
| **120** | **520** | **1050** | **✔** |  | **16** |
| **121** | **1200** | **1200** | **✔** |  | **17** |
| **122** | **134** | **1134** | **✔** |  | **49** |
| **123** | **767** | **1000** | **ND** |  | **12** |
| **124** | **267** | **1100** | **✔** |  | **13** |
| **125** | **750** | **912** | **✔** |  | **14** |
| **126** | **750** | **850** | **✔** |  | **9** |
| **127** | **300** | **700** | **✔** |  | **34** |
| **128** | **800** | **1000** | **✔** |  | **ND** |
| **129** | **867** | **1100** | **✔** |  | **24** |
| **130** | **E** | **E** | **✔** |  | **ND** |
| **131** | **200** | **1000** | **✔** |  | **29** |
| **132** | **767** | **967** | **✔** |  | **4** |
| **133** | **700** | **1100** | **✔** |  | **17** |
| **134** | **680** | **880** | **✔** |  | **18** |
| **135** | **645** | **845** | **✔** |  | **ND** |
| **136** | **1167** | **1500** | **✔** |  | **11** |
| **137** | **580** | **745** | **✔** |  | **1** |
| **138** | **600** | **967** | **✔** |  | **19** |
| **139** | **500** | **1000** | **✔** |  | **18** |
| **140** | **480** | **780** | **✔** |  | **16** |
| **141** | **667** | **900** | **✔** |  | **13** |
| **142** | **845** | **845** | **✔** |  | **ND** |
| **143** | **834** | **1100** | **✔** |  | **2** |
| **144** | **734** | **1067** | **✔** |  | **4** |
| **145** | **920** | **1120** | **✔** |  | **10** |
| **146** | **767** | **900** | **ND** |  | **7** |
| **147** | **785** | **1020** | **✔** |  | **6** |
| **148** | **800** | **867** | **✔** |  | **14** |
| **149** | **450** | **980** | **✔** |  | **18** |
| **150** | **1020** | **1250** | **ND** |  | **6.5** |
| **151** | **820** | **820** | **✔** |  | **7** |
| **152** | **885** | **1050** | **✔** |  | **ND** |
| **153** | **500** | **667** |  | **extra band at 100bp** | **13** |
| **154** | **612** | **912** | **✔** |  | **9** |
| **155** | **E** | **E** | **✔** |  | **ND** |
| **156** | **312** | **780** | **✔** |  | **ND** |
| **157** | **645** | **880** | **✔** |  | **ND** |
| **158** | **712** | **900** | **✔** |  | **ND** |
| **159** | **645** | **812** | **✔** |  | **6** |
| **160** | **785** | **785** | **✔** |  | **1.5** |
| **161** | **E** | **E** | **✔** |  | **ND** |
| **162** | **680** | **745** | **✔** |  | **ND** |
| **163** | **E** | **E** | **✔** |  | **ND** |
| **164** | **E** | **E** | **✔** |  | **ND** |
| **165** | **E** | **E** | **✔** |  | **ND** |
| **166** | **167** | **500** | **✔** |  | **51** |
| **167** | **720** | **920** | **✔** |  | **3** |
| **168** | **583** | **1183** | **✔** |  | **22** |
| **169** | **E** | **E** | **✔** |  | **1** |
| **170** | **1100** | **1134** | **ND** |  | **6** |
| **171** | **634** | **767** | **✔** |  | **9** |
| **172** | **167** | **834** | **✔** |  | **28** |
| **173** | **100** | **1100** | **✔** |  | **55** |
| **174** | **412** | **850** | **✔** |  | **33** |
| **175** | **380** | **780** | **✔** |  | **42** |
| **176** | **834** | **1034** | **✔** |  | **12** |
| **177** | **585** | **1250** | **✔** |  | **14** |
| **178** | **785** | **785** | **✔** |  | **5** |
| **179** | **400** | **834** | **✔** |  | **17** |
| **180** | **100** | **500** |  | **19bp deletion in 3’ flanking region** | **15** |
| **181** | **780** | **980** | **✔** |  | **7** |
| **182** | **334** | **900** | **✔** |  | **20** |
| **183** | **467** | **667** | **✔** |  | **12** |
| **184** | **650** | **850** | **ND** |  | **13** |
| **185** | **700** | **1000** | **✔** |  | **15** |
| **186** | **800** | **1000** | **✔** |  | **8** |
| **187** | **1100** | **1234** | **✔** |  | **10** |
| **188** | **200** | **1000** | **✔** |  | **30** |
| **189** | **834** | **1200** | **✔** |  | **3** |
| **190** | **800** | **867** | **✔** |  | **8** |
| **191** | **1000** | **1200** | **✔** |  | **8** |
| **192** | **720** | **920** | **✔** |  | **19** |
| **193** | **720** | **1020** | **✔** |  | **19** |
| **194** | **567** | **1000** | **✔** |  | **22** |
| **195** | **850** | **1150** | **✔** |  | **3** |
| **196** | **850** | **850** | **✔** |  | **2** |
| **197** | **685** | **1120** | **✔** |  | **6** |
| **198** | **750** | **850** | **✔** |  | **4** |
| **199** | **380** | **520** | **✔** |  | **19** |
| **200** | **720** | **885** | **✔** |  | **18** |
| **201** | **900** | **1200** | **✔** |  | **14** |
| **202** | **450** | **985** | **✔** |  | **16** |
| **203** | **920** | **920** | **✔** |  | **10** |
| **204** | **450** | **985** | **✔** |  | **19** |
| **205** | **850** | **1150** | **✔** |  | **7** |
| **206** | **1185** | **1185** | **✔** |  | **12** |
| **207** | **985** | **1120** | **✔** |  | **12** |
| **208** | **1050** | **1050** | **✔** |  | **8** |
| **209** | **N** | **867** | **✔** |  | **15** |
| **210** | **800** | **1134** | **✔** |  | **6** |
| **211** | **834** | **834** | **✔** |  | **4** |
| **212** | **700** | **800** | **✔** |  | **11** |
| **213** | **834** | **1100** | **ND** |  | **10** |
| **214** | **200** | **1100** | **✔** |  | **36** |
| **215** | **734** | **900** | **✔** |  | **7** |
| **216** | **720** | **720** | **✔** |  | **10** |
| **217** | **400** | **667** | **✔** |  | **11** |
| **218** | **600** | **834** | **✔** |  | **12** |
| **219** | **634** | **1100** | **✔** |  | **10** |
| **220** | **767** | **1134** | **✔** |  | **15** |
| **221** | **700** | **1000** | **✔** |  | **5** |
| **222** | **785** | **850** | **✔** |  | **9** |
| **223** | **700** | **1200** | **✔** |  | **12** |
| **224** | **467** | **967** | **✔** |  | **15** |
| **225** | **567** | **900** | **✔** |  | **13** |
| **226** | **767** | **867** | **✔** |  | **8** |
| **227** | **600** | **1100** | **✔** |  | **17** |
| **228** | **434** | **600** | **✔** |  | **22** |
| **229** | **734** | **900** | **✔** |  | **13** |
| **230** | **667** | **767** | **✔** |  | **3** |
| **231** | **745** | **945** | **✔** |  | **5** |
| **232** | **1080** | **1080** | **✔** |  | **17** |
| **233** | **445** | **780** | **✔** |  | **15** |
| **234** | **780** | **880** | **✔** |  | **7** |
| **235** | **745** | **845** | **✔** |  | **6** |
| **236** | **645** | **780** | **✔** |  | **6** |
| **237** | **412** | **645** | **✔** |  | **14** |
| **238** | **212** | **845** | **✔** |  | **33** |
| **239** | **245** | **912** | **✔** |  | **24** |
| **240** | **780** | **1180** | **✔** |  | **11** |
| **241** | **45** | **745** | **✔** |  | **13** |
| **242** | **650** | **980** | **✔** |  | **6** |
| **243** | **E** | **E** | **✔** |  | **ND** |
| **244** | **E** | **E** | **✔** |  | **ND** |
| **245** | **E** | **E** | **✔** |  | **ND** |

**N = normal allele of undetermined size**

**E = expanded allele of undetermined size**

**C in grey box = carrier status:**

**(F1) 3 and 4 are the parents of proband 2**

**(F2) 5 is a parent of proband 7**

**(F3) 9 and 10 are the parents of proband 11**

**(F4) 18 is a parent of proband 19**

**ND=not determined**

**GAA sizes for samples 89-245 were obtained from the EFACTS database**
